# Optimal Deconvolution of Transcriptional Profiling Data Using Quadratic Programming with Application to Complex Clinical Blood Samples

**DOI:** 10.1371/journal.pone.0027156

**Published:** 2011-11-16

**Authors:** Ting Gong, Nicole Hartmann, Isaac S. Kohane, Volker Brinkmann, Frank Staedtler, Martin Letzkus, Sandrine Bongiovanni, Joseph D. Szustakowski

**Affiliations:** 1 Biomarker Development, Novartis Institutes for BioMedical Research, Cambridge, Massachusetts, United States of America; 2 Biomarker Development, Novartis Institutes for BioMedical Research, Basel, Switzerland; 3 Harvard Medical School, Children's Hospital Medical Center, Boston, Massachusetts, United States of America; 4 Department of Autoimmunity, Transplantation and Inflammation, Novartis Institutes for BioMedical Research, Basel, Switzerland; University of Sheffield, United Kingdom

## Abstract

Large-scale molecular profiling technologies have assisted the identification of disease biomarkers and facilitated the basic understanding of cellular processes. However, samples collected from human subjects in clinical trials possess a level of complexity, arising from multiple cell types, that can obfuscate the analysis of data derived from them. Failure to identify, quantify, and incorporate sources of heterogeneity into an analysis can have widespread and detrimental effects on subsequent statistical studies.

We describe an approach that builds upon a linear latent variable model, in which expression levels from mixed cell populations are modeled as the weighted average of expression from different cell types. We solve these equations using quadratic programming, which efficiently identifies the globally optimal solution while preserving non-negativity of the fraction of the cells. We applied our method to various existing platforms to estimate proportions of different pure cell or tissue types and gene expression profilings of distinct phenotypes, with a focus on complex samples collected in clinical trials.

We tested our methods on several well controlled benchmark data sets with known mixing fractions of pure cell or tissue types and mRNA expression profiling data from samples collected in a clinical trial. Accurate agreement between predicted and actual mixing fractions was observed. In addition, our method was able to predict mixing fractions for more than ten species of circulating cells and to provide accurate estimates for relatively rare cell types (<10% total population). Furthermore, accurate changes in leukocyte trafficking associated with Fingolomid (FTY720) treatment were identified that were consistent with previous results generated by both cell counts and flow cytometry. These data suggest that our method can solve one of the open questions regarding the analysis of complex transcriptional data: namely, how to identify the optimal mixing fractions in a given experiment.

## Introduction

With its capacity for simultaneous monitoring of the transcriptional state of thousands of genes, high-throughput transcriptional profiling using DNA microarrays has provided investigators with a unique opportunity for genome-wide regulatory analysis in clinic trials and biomarker identification. Molecular analysis of cells in their native tissue environment provides the most accurate picture of the *in vivo* disease state [1]. The complicated structures of tissues and cellular environments, composed of large numbers of disparate yet interacting cell populations, makes this difficult. RNA prepared from heterogeneous tissue samples might contain only a fraction of the total cell subpopulation of interest [2]. Consequently, the expression signal of any gene detected directly from a complex sample is a convolution of expressions of all present cell types. Therefore, if tissues or cells are used without consideration of such a mixing phenomenon, measurement of differential gene expression will certainly be confounded by the heterogeneous cell populations [3], [4]. Similarily, heterogeneity of cell populations across different samples could drown out the variability resulting from other, perhaps more relevant differences between samples [5].

There are several approaches used to identify changes in gene expression that occur in different cellular compartments within tissues or tumors comprised of multiple cell types. Microdissection techniques that might allow a purer sampling of cells from fresh tumor specimens is time-consuming and requires an amplification of the sample that could distort transcriptional profiles [6]. Blood cell-type subset composition can be measured by complete blood counts (CBCs). CBCs typically offer a fixed, low resolution survey of circulating cell populations. For example, a typical CBC will provide one measurement that describes all circulating lymphocytes. Such data can not be used to tease apart contributions from important cell populations including CD4+ and CD8+ T-cells, B-cells, or Natural Killer (NK) cells, each of which is derived from a distinct lineage and carries out a different immunological purpose. Nevertheless, it has been demonstrated that the incorporation of CBC measurements helps ellucidate meaningful transcriptional signals in blood [3].

The inversion of sample heterogeneity can be facilitated by providing accurate estimates of the mixing percentages of different cell types through computational deconvolution. Since computational dissection does not require microdissection of all samples or change of routine biological protocols, several authors have tried to answer whether it is possible to decompose the DNA microarray data from a cell population to survey the proportions of different cell types, by treating specific transcriptional patterns in DNA microarray data as cell-type-specific markers through computational methods [3], [5], [7], [8], [9], [10], [11]. Lu *et al*. pioneered the application of a simulated annealing-based algorithm to identify the proportions of cells [11]. Abbas *et al*. [8] first applied microarray deconvolution for measuring proportions of cell types in blood samples and employed the results to study immune disease. Quon uses Latent Dirichlet Allocation (LDA) to implement the deconvolution strategy in conjunction with digital high-throughput sequencing data [9]. Very recently, Shen-Orr *et al*. described cell-type-specific significance analysis of microarrays (csSAM) for analyzing differential gene expression for each cell type in a biological sample by incorporating heterogeneity in gene expression [3]. Nevertheless, previously developed approaches for tackling heterogeneity in transcriptional profiling data from complex samples have several drawbacks which we aim to address and alleviate in this study. Some methods can only be applied to two-source systems; that is to say, complex mixtures composed of only two tissue or cell types [7], which is not practical for application to more complex samples. Other approaches have been reported to deconvolute heterogeneous expression profiles into their individual component profiles and thereby infer the mixing proportions. However, these do not guarantee a globally optimal solution, nor do they guarantee physically meaningful solutions. These approaches use heuristic methods that non-deterministically identify local optima [5], [11], or require *ad hoc* post-processing to eliminate non-physical results such as negative mixing fractions [8].

What we sought to demonstrate here was an *in silico* approach to deconvolute gene expression profiles obtained from heterogeneous clnicial samples into cell-type-specific patterns when the mixing matrix is unknown. We developed an approach built upon linear latent variable models that efficiently identifies the globally optimal solution in the least squares sense. Moreover, our approach explicitly incorporated physical constraints, specifically the mixing weights were required to be non-negative and sum to one, and therefore generated results that can be directly interpreted as mRNA mixing fractions. Technically, we employed a supervised selection of cell-type-specific genes to provide a basis that described the transcriptional state of “pure” cell populations. These cell-type-specific transcripts were then used to deconvolute the samples of interest using a quadratic programming technique that was highly efficient, providing directly interpretable results (i.e., the mixing fractions), and guaranteed to find the globally optimal solution. The results demonstrated that our method was able to accurately predict mixing fractions for more than ten species of circulating cells, and was even able to provide accurate estimates for relatively rare cell types.

## Results

We implemented our procedure for estimating fractions of different cell types in multiple gene expression data sets. First we assessed the utility of our method by applying it to three well controlled benchmark data sets with known mixing fractions. Satisfied that our approach worked, we then applied it to more challenging mRNA expression profiling data from human blood samples collected as part of a clinical trial.

### Proof of Concept: Deconvolution Accurately Predicts Mixing Fractions

#### Datasets

We used three benchmark datasets as proof of concept experiments. In the first experiment, tissues used for microarray analyses included independent, triplicate pools of blood and breast tissue samples from female adults. Double standed cDNA synthesis and labeling was carried out with 5 µg of total RNA, each sample was hybridized to Human Genome 133 Plus 2.0 GeneChips as specified by the manufacturer and the resulting CEL files were processed by Robust Multiarray Average (RMA) normalization [12] and scaled to a 2% trimmed mean of 150. Six purified reference sample data files and nine other mixtures included RNA from each of the two tissues at varying proportions were summarized in Table 1. The array data can be accessed via Gene Expression Omnibus (GEO), GSE 29832.

**Table 1:** Experimental design for blood *vs*. breast microarray experiment.

Tissue Type	% Blood mRNA	% Breast mRNA	# Replicates
Pure	0%	100%	3
Mixed	33%	67%	6
Mixed	67%	33%	3
Pure	100%	0%	3

RNA derived from 15 female adults were homogenaized, extracted and mixed in 4 different proportions, two of which are each of the tissues in isolate (100% blood and 100% breast).

In the second experiment, we employed the MAQC Rat Toxicogenomics Dataset [13] which includes RNA samples using Rat Genome 230 2.0 GeneChips. The RNA derived from rat liver and kidney bio-specimens from a single rat was mixed in four different proportions, two of which were from each of the tissues in isolate (100% liver and 100% kidney). The two other mixtures included RNA from each of the two tissues are 75∶25/25∶75 respectively (Table S1). MIAME-compliant array data can be accessed via Gene Expression Omnibus (GEO), GSE5350.

For the last benchmark dataset, we used rat liver and brain as described in [3]. Each sample was hybridized to rat-specific RAE230_2 whole-genome expression arrays (Affymetrix), and the resulting CEL files were processed by RMA normalization for deconvolution. Each of the samples was analyzed in triplicate. The detailed mixture information is shown in Table S2. The microarray data used in this study (series number GSE19830) are available at NCBI-GEO [14].

#### Expression Signatures

Microarray expression data were used to generate cell-type-specific gene lists through pairwise comparisons of expression between all pure samples as described in *Materials and Methods*. Statistical associations between GO annotation and lists of differentially expressed genes were identified using MetaCore™ [15]. We applied the False Discovery Rate (FDR) multiple testing correction [15] and applied a final cutoff of FDR adjusted *p*<0.05 to identify statistically significant associations.

Inspection of annotation of identified gene list in blood *vs*. breast cell line data confirmed this approach returned known cell specific transcripts, pathways, and biological processes. The gene signature included genes whose expression is specific for blood specific genes (BANK1, BCL11B), breast specific genes (ERBB3, CA12, CCND1, ESR1) (Table S3). And all these genes are enriched in cell cycle control, role APC in cell cycle regulation, the metaphase checkpoint; human Cell-cycle/CDKN1A Mediated Pathway and their enriched GO categories included mitosis (biological process), M phase of mitotic cell cycle (biological process), M phase (biological process), cytokinesis (biological process) and cell division (biological process). These findings support the validity of this approach to identify cell-type-specific genes. Detailed annotations and Gene Ontology over-representation analyses are shown in Table S3.

### Expression Deconvolution on Cell Line Mixing Experiments

First, we measured the accuracy of our method with three benchmark experiments where known proportions of different tissues or cells are mixed, assayed on expression microarrays, and computationally separated.

**Figure 1:**
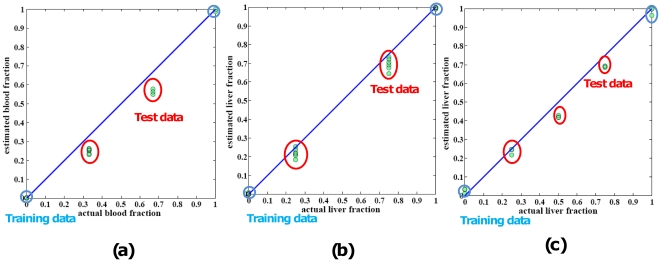
Statistical deconvolution of complex tissues yields accurate estimates of pure tissue fractions. Plotting of proportions of cell lines determined from deconvolution (y axis) *vs*. proportions of the cell lines actually mixed (x axis) shows strong congruity. (a) Proportions of blood cells determined by deconvolution are similar to proportions determined by actual blood fraction. Diagonal lines are y = x, shown for reference, highlighting the agreement between the two methods. The training data in blue circles are from pure reference samples. The test data are from mixed samples with various mixing proportions. (b) Proportions of liver fraction determined by deconvolution are similar to actual liver fraction. (c) Proportions of liver cell lines determined from deconvolution *vs*. proportions of the cell lines actually mixed are shown a high consistency in rat liver *vs*. brain dataset.

In each case, we generated gene signatures by analyzing the data from the “pure” samples (Training Data) and then applied these signatures into our approach to estimate the mixing fractions for the complex samples (Test Data). The results of the first mixtures - blood *vs*. breast are as depicted to the Fig. 1(a). The congruence between our predictions and the actual mixing fractions suggests the validity of this deconvolution approach. Secondly, we deconvoluted rat liver and kidney mixture dataset. As expected, this algorithm also correctly estimated the composition of each of the 12 samples as consisting entirely of its appropriate corresponding cell types (Fig. 1(b)). In the third experiments, expression deconvolution was performed on data sets of the mixture of rat liver and brain (Fig. 1(c)). These estimates closely paralleled changes in component sizes that were observed by known fractions, thereby confirming the validity of this approach.

### Deconvolution of Circulating Cells from Whole Blood Samples

To test the utility of our algorithm to track clinically relevant changes in blood populations, we applied our method to expression profiling data generated for whole-blood samples collected from Multiple Sclerosis patients (MS) treated with Fingolimod (FTY720), a novel immunomodulator. Fingolomid is a structural analog of sphingosine that, in its phosphorylated form (FTY720-P), antagonizes S1P1 receptors expressed on the surface of lymphocytes. This in turn prevents the egress of lymphocytes from the lymph nodes, thereby impacting the trafficking of lymphocytes in the circulation [16]. It was previously shown that Fingolomid preferentially reduces the number of circulating CD4+ and CD8+ T-cells in human subjects [16].

**Figure 2:**
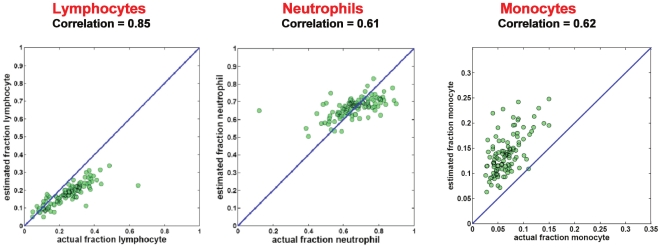
Comparison of CBC data and statistical deconvolution in whole blood samples. Determination in whole blood samples of relative abundance of total lymphocytes, neutrophils, or monocytes by CBC compared to determination of relative abundance by deconvolution. Each green dot here corresponds to one sample in the dataset. Diagonal lines are y = x, shown for reference, highlighting the agreement between the two methods.

Blood is a particularly complex tissue type, with over a dozen distinct cell types that can vary in frequency up to 10∼20-fold between healthy individuals [3]. We applied our method to whole blood samples, using previously published signatures [8] for 17 circulating cell types (Table S4). We aggregated our predictions within three major cell types (lymphocytes, monocytes and neutrophils) to facilitate direct comparison to the CBC results. Agreement between our predictions and measured values was excellent (Fig. 2), with Pearson correlation coefficients ranging from 0.61 to 0.85. Agreement between predicted and measured values was greatest for lymphocytes, which is notable due to the complexity of sub-populations present in this fraction. In contrast, previous attempts to deconvolute blood samples using the same signatures have only achieved lower correlations against CBC data (0.52 and below) [8]. As depicted in Fig. 2, agreement between predicted and actual cell fractions shows good correlation, but deviates from the diagonal. This can be attributed to intrinsic differences in mRNA amounts per cell type and extrinsic differences in mRNA yield. These deviations are linear in nature, and therefore would not impact most downstream applications.

**Figure 3:**
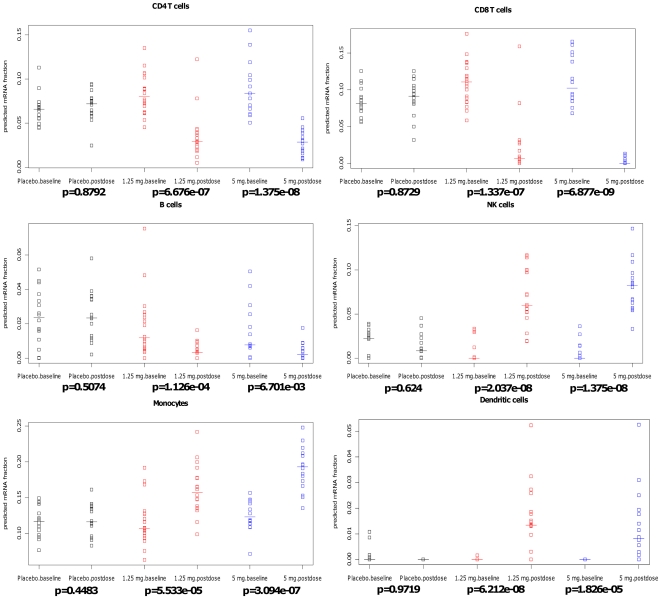
Estimated fractions for several circulating cell populations. Strip charts display relating quality of CD4+ cells/CD8+/B cells/NK cells/Monocytes/Dendritic cells. The data are stratified in three different subgroups: placebo (black), low dose: 1.25 mg/day (red) and high dose: 5 mg/day (blue). Data points are from each donor. Y axis is the estimated mRNA fraction. P-values are calculated by Wilcoxon's Signed Rank test.

Inspection of the predicted mRNA fractions revealed that our method was able to dissect the lymphocyte population and track changes in specific populations induced by Fingolomid. Fig. 3 depicts a detailed breakdown of our predictions, stratified on treatment group and time point. Our method correctly identified reduction in circulating CD4+, CD8+, and B-cells following Fingolomid treatment. Reductions relative to baseline were significant for both treatment arms (*p*<0.01, Wilcoxon ranked sum test). Several other populations had increases in their relative predicted proportions in the treated subjects. Specifically, the predicted relative abundance of monocytes, NK cells and dendritic cells increased following Fingolomid treatment. These populations are not sequestered in the lymph nodes following treatment by Fingolomid, so their absolute numbers in circulation remain unchanged. Because microarray data is inherently semi-quantitative, we are only able to determine the relative abundance of each cell type in a sample. Consequently, the relative abundance of the cells appears to increase concomitant with the Fingolomid-induced reduction in other lymphocytes. Notably, none of the populations demonstrated changes in the placebo-treated subjects (*p*>0.4), which suggested that our approach was capable of a high degree of specificity even in complex, highly variable data sets.

### Comparison to Other Methods

All surveyed microarray deconvolution methods make use of a system similar to that described in equation (1), **X** = **AS**. They differ substantially, however in how they dissect this system of equations, their optimization methods, and other important details. A summary of the methods surveyed here, and their main characteristics can be found in Table 2.

**Table 2:** Summary of current major deconvolution methods.

	Decoupled/simultaneously estimation	Deterministic/probabilistic	Global/local optimal solution	Non-negative constraint	Related to sample size	Source code available
Our method	Decoupled, estimate **A**	deterministic	global optimal solution by quadratic programming	Yes	No	-
Abbas, plus ONE, 2009	Decoupled, estimate **A**	deterministic	Local	No	No	No
Shen-Orr, Nature Methods, 2010	Decoupled, estimate **S**	deterministic	Global	No	No	Yes
Repsilber, BMC bioinformatics, 2010	Simultaneously estimate **A** and **S**	deterministic	Local	Yes	No	Yes
Erkkila, Bioinfoamtics, 2010	Simultaneously estimate **A** and **S**	probabilistic	Local	Yes (implicitly)	Yes	Yes
Stuart, PNAS, 2004	Decoupled, estimate **S**	deterministic	Global	No	No	No
Lu, PNAS, 2003	Decoupled, estimate **A**	probabilistic, simulated annealing-based algorithm	the probability that the simulated annealing algorithm terminates with the global optimal solution approaches 1 as the annealing schedule is extended	Yes (implicitly)	No	The software link no longer works

These methods approach equation (1) in one of three ways: (A) Given microarray data **X** and mixing fractions **A**, estimate the basis matrix **S**. Shen-Orr *et al.* [3] used this approach to combine cell count and microarray data as input for further analysis to identify disease-associated transcriptional disregulation. (B) Given microarray data **X** and basis matrix **S**, estimate the mixing fractions **A**. Our approach falls into this category. (C) Given microarray data **X**, simultaneously estimate the mixing fractions **A** and basis matrix **S**. This approach is unsupervised. Consequently, such methods require prior information to initialize the optimization [17], use non-deterministic optimizers that can become trapped in local minima [18], must label the pure cell types in post-processing steps [18], and vary in performance depending on the amount of input data [18].

Immune cell-specific expression is a critical indication of a gene's role in the immune response [19]. Fortunately, a compendium of microarray expression data for human genes from key immune cell types has been compiled [19], making it possible to supervise the decomposition with respect to these known primary immune cell types and these subsets of genes. As a demonstration, we applied our method, and two methods with available source code [18] and [17] to two benchmark data sets. These methods are designed to solve the more general and challenging problem (category (C) above) of solving for cell signatures and cell fractions simultaneously. Method [18] operates in a completely unsupervised fashion, wheres method [17] requires an initial estimate of the cell fractions. A direct comparison of the performance of these methods is challenging, however we believe it provides some insights into the relative strengths and weaknesses of each, and helps to assess the importance of prior biological knowledge when deconvoluting complex data. Our first benchmarks were run on the blood/breast data set. The method of Erkkila *et al.* [17] requires initial estimates of the mixing fractions. To test this method, we provided it with initial estimates based on the known fractions with Gaussian noise added at 20dB (i.e. 100∶1 signal to noise ratio (SNR)). A second set of benchmarks were run on the 24 whole-blood microarrays described in [3], and compared to the published Complete Blood Counts (CBCs) to assess accuracy. Again, we seeded method [17] with random numbers, CBCs with 20dB Gaussian noise (100∶1 SNR), and CBCs with 10dB Gaussian noise (10∶1 SNR) respectively. Results are presented in Table 3. For the simple blood/breast system, all methods performed well; performance for our method and Repsilber *et al.* 's [18] was similar (correlation >0.99) and slighlty better than the performance of Erkkila *et al.* 's approach [17] (correlation >0.96). For the more complex blood sample, our method and Repsilber *et al.* 's performed similarly for neutrophils, whereas ours performed substantially better for lymphocytes and monocyte. Erkkila *et al.* 's approach performed better than the other methods when seeded with the actual CBC values with mild noise (20 dB); however its performance degraded for more realistic tests with added noise. At 10 dB noise its performance drops but is similar to the results of our method; in the absence of prior information (i.e. seeded with random estimates) it could not find any solutions. These benchmarks indicate that deconvolution performance varies by the complexity of the experimental system, and the availability of prior biological knowledge. Our method, when fed with cell-type-specific transcriptional signatures, appears to perform well across a number of different biological systems of varying complexity. For the more general case in which both cell-type-specific signatures and cell fractions are not known, the performance of available methods varies substantially. For simple systems with few cell types, all tested methods perform well. For more complex systems, the use of accurate prior knowledge in the form of signatures (our method) or accurate cell fraction estimates (Erkkila *et al.* 's method [17]) results in better performance. The results from Repsilber *el al*. 's method [18] indicates that reasonable cell fraction estimates are still possible in the absence of prior knowledge. Taken together, these results suggest that (1) there is no one size fits all solution to this problem and (2) one should take advantage of any available prior biological knowledge when attempting to deconvolute transcriptional data.

**Table 3:** The comparison of deconvolution methods on cell line data and Shen-Orr *et al.*'s 24 whole-blood microarray data.

Methods	Breast/blood cell line data	Human whole-blood gene expression array data from kidney transplant recipients		
		Neutrophils	Lymphocytes	Monocytes
Our method	0.9912	0.7198	0.6926	0.6492
Repsilber *et al.*, BMC bioinformatics, 2010	0.9901	0.7092	0.4764	0.2783
Erkkila *et al.*, Bioinformatics, 2010	-	-0.1135**^a^**	0.2926**^a^**	0.1147**^a^**
Erkkila *et al.*, Bioinformatics, 2010	-	0.6324**^b^**	0.7381**^b^**	0.5359**^b^**
Erkkila *et al.*, Bioinformatics, 2010	0.9665**^c^**	0.955**^c^**	0.9094**^c^**	0.8865**^c^**

The numbers of the table showed the correlation coefficients between predicted and measured values for mixing proportions. For cell line data, we initialized the mixing matrix for Erkkila *et al*.'s approach with measured CBC fractions added 20 dB noise**^c^**. For Shen-Orr *et al*.'s data, we provided three different kinds of prior knowledge for the initialization of mixing matrix for Erkkila *et al*.'s approach: random numbers from normal (or Gaussian) distribution as the mixing fraction**^a^**, measured fractions with 10 dB noise**^b^** and measured fractions with 20 dB noise**^c^**. We aggregated our predictions within three major cell types (neutrophils, lymphocytes and monocytes) to direct compare to the CBC results.

### Robustness of the Gene Signature Selection

The foundation for this approach is the identification of a set of signatures that are generally representative of the cell types of interest. Any errors or uncertainities introduced in the design of this basis matrix could propogate through the analysis and impact the final results. Our approach leverages the thousands of expression level measurements made on each microarray to define a system of linear equations that are overdetermined, and can be optimally solved globally via quadratic programming. Taken together, this strategy should be robust to small deviations in the basis matrix; it uses many measurements of probes on the chip (*j*>100) to estimate a small number of parameters in the linear system (*n*<20), so errors in any one measurement should have only a minimal effect on the final estimations. We performed several simulations using the blood/breast data to verify the robustness of our approach to fluctuations in the construction of the basis matrix.

**Figure 4:**
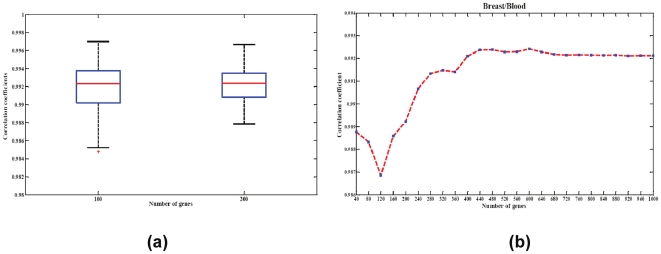
Robustness of the signature matrix. (a) Boxplot displaying robustness of chosen signature matrix to gene content. The correlation coefficient distribution (Y axis) is depicted for signatures composed of 100 or 200 randomly selected differentially expressed probesets. (b) Deconvolution performance across a range of signature sizes. The experiment is conducted by increasing the number of cell-type-specific gene probes step-wisely from 40 to 1000.

For the first simulation, we sought to address the impact of the selection of differentially expressed genes inlcuded in the basis matrix. There were 1320 differentially expressed probesets identified in the blood/breast experiment. We randomly selected either 100 or 200 probesets from these 1320 for inclusion in the basis matrix and then estimated the mixing fractions using the new basis matrices. This procedure was repeated 100 times. Results are depicted in Fig. 4 panel (a). As expected, results were robust to the precise selection of differentially expressed genes, with correlations between estimated and actual fractions above 0.99 for almost all simulated matrices.

We then examined the accuracy of our approach by increasing the number of cell-type-specific gene probes stepwise from 40 to 1000. The correlation coefficients plot (Fig. 4(b)) shows that our approach accurately estimated the mixing proportions as long as the basis matrix includes at least 240 probesets. The estimation could steadily achieve the correlation coefficient above 0.99.

**Figure 5:**
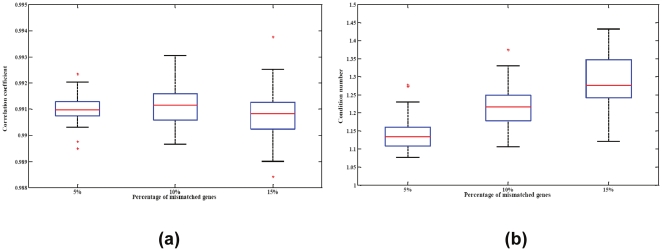
Stability of the signature matrix. (a) Boxplot displaying the stability of chosen signature matrix. The chosen signatures are distorted by randomly selecting 5, 10, or 15 percent of its genes and randomly modulating their values with 2 fold changes. The distribution of correlations between actual mixing fractions and fractions estimated using these signatures is depicted. (b) Condition number of the basis matrix with respect to the percentage of simulated differentially expressed genes in the basis matrix.

Finally, the basis matrices might also be challenged through the introduction of biological variability. Ideally, one would like to construct basis matrices from training experiments that are as similar as possible to the eventual test conditions. This however is not always possible. Clinical samples are precious commodities, cell-sorting techniques can be cumbersome or costly, and real-world applications often involve systems perturbed by disease or other interventions. We conducted several simulations to further evaluate the generalizability of our approach when genes selected for the basis matrix were differentially expressed in the test systems. We randomly selected 5, 10, or 15 percent of the genes in the basis matrix and altered their values by factors of +/−2 fold and +/−5 fold. This process was repeated 100 times, and we compared the estimates using the modulated basis matrices to the actual fractions. The two-fold changes simulation results are shown in Fig. 5, while the five-fold change simulation experiments are presented in Fig. S4. Fig. 5(a) illustrates that our algorithm still achieves very significant accuracy with the correlation coefficients between the estimated and measured proportions above 0.99. This is true even in the extreme case where 15% of the genes in the basis matrix are changed.

These three sets of simulations demonstrate that our approach – an overdetermined system of equations coupled to efficient global optimization – is robust against the kind of biological and technical noise we expect to see in real world applicaitons.

## Discussion

We have developed a novel computational approach for deconvoluting mRNA expression profiling data from complex samples into contributions from an aribirtrary number of cell types for which prior biological knowledge is available. We built upon the well accepted practice of describing such data as a system of linear equations through the introduction of a least squares solution with equalities and inequalities that can be optimally solved via quadratic programming. The use of quadratic programming has several advantages over methods previously used to address this problem. Specifically, this approach allows for the explicit modeling of physical constraints in both the description of the problem as well as its solution. Application of equalities and inequalities in turn enables direct interpreation of the results as mRNA proportions. In addition, the introduction of quadratic programming as an optimizer provides a computationally efficient algorithm that gurantees the identification of a globally optimal solution to the system of equations. The introduction of quadratic programming should close one of the open questions in this field, namely how best to solve the system of equations used to represent complex microarray data. Going forward, we believe there is still substantial room to improve other aspects of this framework. As examples, the generation of cell-specific signatures is still a largely heuristic endeavor, and the lower limits of detection of rare cell types remains largely uncharacterized.

Our approach yielded predictions with excellent agreement to measured values across a number of simple controlled mixing experiments. Through the use of accurate transcriptional signatures for various circulating cell types, we have demonstrated that our method is capable of generating accurate predictions of even rare cell types in complex blood samples. Moreoever, this approach has clearly demonstrated an ability to track clinically relevant changes in blood populations that would be missed in standard CBCs.

This work provides a critical step toward the improved analysis of transcriptional data derived from complex clinical samples. In the case decribed here, our method was able to accurately predict drug-induced changes in lymphocyte trafficking based solely on mRNA expression profiling data. These and other changes in cicrulating cell populations in clinical settings are of sufficient magnitude to dominate the signals measured via transcriptional profiling, and would color any analysis that does not account for them. Previous work has suggested that it is possible to dissect cell-specific transctiptional changes *in silico* [3] using CBC data as a guide post. Our methodology and results allow for a much finer grained view of cell heterogeneity that should enable more precise *in silico* dissection.

Looking forward, we see several natural extensions of our method. The rapid adoption of Next Generation Sequencing platforms (NGS) promises the delivery of increasingly higher resolution views of the transcriptome. Data from such RNA-Seq experiments is already providing more exquisite views of low-abundance transcripts and alternative splicing [20]. Identification of new transcriptional species is likely to make deconvolution more sensitive and accurate. The ability to detect low-abundance transcripts should allow us to detect rarer cell populations, while the broader sampling of the transcriptome should aid in the idenfitication of cell-type-specific isoforms that will more precisiely delineate closely related cell populations. This is likely to be of great importance in the application to blood samples, where increased resolution and sensitivity would allow us to differentiate between clinically relevant subpopulations (e.g. Th1, Th2, and Th17 CD4+ T-cells). Another natural example would be application to metagenomics experiments to explicitly estimate the relative abundance of various microorganisms based on the abundance of their DNA in a sample.

In general, the application of highly sensitive, high throughput experimental technologies to complex biological samples will require increasing sophistication in the way that we think about and analyze our data. In some cases, this complexity has the potential to obfuscate relevant phenomenon if not addressed. In others, accurately estimating the complexity itself can be a useful endpoint. The approach we introduced here represents one specific application of a general framework for explicitly handling such complexities. The mathematical underpinnings and optimization algorithm are agnostic to the details of the biological system, and are generalizable to other data types that can be described via a system of overdetermined linear equations.

## Materials and Methods

### Patients and Whole Blood Smaples

Whole blood transcriptional analysis was performed as part of a clinical trial [21] (CFTY720D2201, a double-blind, randomized, placebo-controlled, parallel-group, multicenter study evaluating the safety, tolerability and effect on MRI lesion parameters of Fingolomid vs. placebo in patients with relapsing multiple sclerosis) (ClinicalTrials.gov identifier NCT00333138). Patients meeting pre-defined disease criteria were treated with Fingolomid at one of two doses (5 mg/day, 1.25 mg/day) or with placebo [16]. The study adhered to the International Conference on Harmonization Guidelines for Good Clinical Practice and was conducted in accordance with the Declaration of Helsinki [22], [23]. All patients gave written informed consent. Characteristics of patients are given in [16]. Whole blood samples were collected in PAXGene tubes for cDNA microarray analysis at baseline (pre-treatment) and at six months after treatment commenced. Samples were then analyzed as described above. The data were also pre-processed using RMA [12] and scaled to a 2% trimmed mean of 150.

### Latent Variable Model

Estimating the proportions of different cell types is based upon a latent variable model framework [24], [25]:
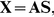
(1)where **X** is the microarray data from complex biological samples, **A** is the set of unknown proportions of the cellular constituents of **X**, and **S** is the known matrix of expression levels of the genes in all the cellular constituents of **X**.

Based on this model, we will first describe how we modeled the total expression signal of each microarray probe as the sum of the expression signals of its constituent parts in each mixture sample and solve it in constrained linear least-squares problems. We will then describe the identification of expression signatures using Limma (Linear Models for Microarray Data) [26] for differential expression analysis and how to estimate the number of expression signatures through condition number of the signature matrix.

### Computational Deconvolution by Linear Least-square Problems

Expression deconvolution, which takes advantage of the linear latent model to represent the original expression signals as a mixture of each compartment signal, was performed on linear, untransformed data as follows. Starting from Eq. (1), the expression level *x_jk_* of gene *j* in a sample *k* is the average of cell type expectations, *s_ij_*, weighted by cell type fractions *a_ki_*:
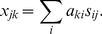
(2)For one probeset, we had many more unknown fractions of mRNA (*a_ki_*) in the sample than known expression level measured on the chip (*x_jk_*), so the system was underdetermined.

For multiple probesets, we could extend this to a system of linear equations:
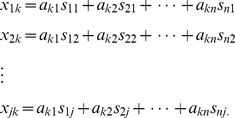
(3)When *j>n* (more probesets than cell types), this system of equations is over determined. Physical constraints could be explicitly added to this system. Microarray data sets are inherently closed. Consequently, we were limited to estimating the proportions of mRNA present from each cell type, and these proportions must sum to one: 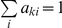. In addition, to insure a physical solution, we required that all mRNA fractions must be non-negative: 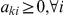.

Ideally, we would like to find mRNA fractions (*a_ki_*) that satisfy: 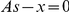. We were unlikely to find such solutions in noisy biological systems. We could, however, find an optimal (*a_ki_*) that minimizes the residuals for 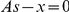 in the least squares sense:
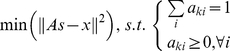
(4)where the coefficient *a_ki_* is a scalar parameter between 0 and 1 to represent the fraction of cell subtype. When linear non-negative inequalities and equalities were given, Eq. (4) could be solved with quadratic programming [27]. We solved this series of equations using the lsqlin function in MATLAB.

It should be noted that this approach has been previously used for deconvoluting populations in complex biological samples, albeit in a completely different setting. Specifically, Mackey *et al.* introduced this approach to successfully estimate the contributions of different phytoplankton classes in oceanic samples based on HPLC measurements of various pigment concentrations [28]. This method is very general and could easily be applied to other data types as well (see *Discussion*). It also has several clear advantages over approaches reported elsewhere. Explicit incorporation of the non-negativity constraint allows clear physical meanings for the solution, which can be directly interpreted as mRNA fractions. Therefore, we do not need to remove the lowest negative coefficient from the equation as in [8], or apply an iterative approach of the solution until all coefficients were nonnegative [5], [11]. Moreover, this system satisfies the criteria necessary to be solvable by quadratic programming, which therefore guarantees a globally optimal solution. Finally, quadratic programming routines are readily available and highly optimized. Solutions even for experiments with hundreds or thousands of samples can be rapidly and efficiently identified on a standard computer workstation.

### Expression signature identification

Expression signatures of homogeneous samples of cells are critical to model the cellular composition of complex tissues. Such signatures provide prior biological knowledge about the “baseline” physiological condition of each cell type. On balance, it is assumed that the baseline condition is represented in complex environments. Generally, many genes remain unchanged across different phenotypes or phenotypic changes [29], [30]; only a subset of the entire gene set potentially discriminates between cell types and may be used to estimate the mixing parameters and represent the pure signals. Hence, only those genes that are able to differentiate cell types of interest are useful as a basis set for microarray deconvolution.

We further reasoned that the expression profiles for high and low abundance genes could fall outside of the linear range of the microarrays, especially in artificial cell line experiments. We observed that there are huge fold changes between different tissues, and therefore only included the genes with the expression value within the range of 0.1∼5000.

The probesets comprising the basis for deconvolution were determined as follows. First, the differential expression of each gene for different tissues or phenotypes was assessed by linear modeling and empirical Bayes methods using Limma (version 3.2.3, [26]) from the Bioconductor project [29]. Genes with an adjusted *p*-value (FDR)<1e-5 were retained for further evaluation. In the next step, we wanted to adjust the number of genes included in the signature to derive a high performance basis matrix that would be attributable to the estimated proportions. Following [8], probesets were ranked by their degree of differential expression according to the absolute t-statistic, and a complete set of matrices comprised of different quantities of the most differentially-expressed probesets was tested by comparing the results of each matrix to the known mixture fractions. A matrix's condition number estimates the sensitivity of a system of linear equations to errors in the data. Consistent with [8], we also observed that the condition number tracked with the accuracy of predictions in a largely continuous fashion (Fig. S1(a)). Additional plots of condition number as a function of matrix size for the liver/kidney and liver/brain experiments are provided in Figs S2 and S3 and their detailed gene lists are in Table S3. These systems had optimal matrices with 210 and 160 probesets, respectively. To test the feasibility of using the condition number as an appropriate selection marker to generate baseline transcriptomes representing genome-wide profiles for different tissues, we did the following experiments.

We validated the ‘optimal’ number of expression signatures in terms of condition number [8] by testing the relationship between the goodness of fitting and different quantities of the most differentially-expressed probesets. Overall, experimentally measured root mean square deviation (RMSD) between the estimated fractions and the actual fractions correlated very closely with how well conditioned (i.e., condition number) each matrix (Fig. S1 (b), Fig. S2 (b) and Fig. S3 (b)). And the RMSD of the fitting residual also had high correlation with the condition number (Fig. S1 (c), Fig. S2 (c) and Fig. S3 (c)). When we selected the ‘optimal’ number of expression signature, the slope of the RMSD of the fitting residual began to gently ease off. All these results supported us to select condition number as a high-fidelity marker for the ability of a basis matrix to accurately deconvolute the mixtures.

In this manner, we obtained optimized size of expression signatures for cell-type-specific genes from each purified reference sample and averaged across samples obtained from the same cell or tissue type. These signatures were taken as estimates of basal expression for computationally deconvolution of mixed samples.

## Supporting Information

Figure S1 **Condition number of signature basis matrix varies with number of probesets included.** (a) Function of the condition number *vs*. the number of probesets from the gene signature was characterized in blood and breast mixture cell lines. The local minima of condition number is shown in green line and the corresponding number of genes was selected as the ‘optimal’ number of expression signature; (b) Root mean square deviation (RMSD) between the estimated fractions and the actual fractions showed clear patterns to support the ‘optimal’ number of expression signature selected in (a). To the right of the green line, the RMSD almost formed a horizontal line with minor oscillations, suggesting that increasing the number of genes would not increase the accuracy of the deconvolution estimates. (c) The RMSD of the fitting residual also had high correlation with the condition number. This correlation is weaker when selecting more than the ‘optimal’ number of genes (shown in green line here).
(TIF)

Figure S2 **Condition number varies with the number of probesets included in liver/kidney signatures.** (a) Function of the condition number *vs*. the number of probesets from the gene signature was characterized in rat liver and kidney mixture cell lines. The local minima of condition number is shown in green line and the corresponding number of genes was selected as the ‘optimal’ number of expression signature; (b) shows the relationship between the RMSD of the estimated fractions and the number of genes in basis matrix; (c) is the plot of the RMSD of the fitting residual *vs*. the number of genes in basis matrix.
(TIF)

Figure S3 **Condition number varies with the number of probesets included in liver/brain signatures.** (a) Function of the condition number vs. the number of probesets from the gene signature was characterized in rat liver and brain mixture cell lines. The local minima of condition number is shown in green line and the corresponding number of genes was selected as the ‘optimal’ number of expression signature; (b) shows the relationship between the RMSD of the estimated fractions and the number of genes in basis matrix; (c) is the plot of the RMSD of the fitting residual *vs*. the number of genes in basis matrix.
(TIF)

Figure S4 **Stability of chosen signature matrix.** (a) Boxplot displaying the stability of chosen signature matrix. The chosen signatures are distorted by randomly selecting 5, 10, or 15 percent of its genes and randomly modulating their values with 5 fold changes. The distribution of correlations between actual mixing fractions and fractions estimated using these signatures is depicted. (b) Condition number of the basis matrix with respect to the percentage of simulated differentially expressed genes in the basis matrix.
(TIF)

Table S1 **Experimental design for rat liver** ***vs*** **. kidney microarray experiment.**
(DOC)

Table S2 **Experimental design for rat brain vs. liver microarray experiment.**
(DOC)

Table S3 **Gene Annotation for blood vs. breast, liver vs. kidney and liver vs. brain experiments.**
(XLS)

Table S4 **Leukocyte types used as the basis for whole blood deconvolution.**
(DOC)
