# Supplementary figures and images for: Optimal Deconvolution of Transcriptional Profiling Data Using Quadratic Programming with Application to Complex Clinical Blood Samples
Source: PLoS One. 2011 Nov 16;6(11):e27156. doi: 10.1371/journal.pone.0027156 (PMC3217948; doi:10.1371/journal.pone.0027156)

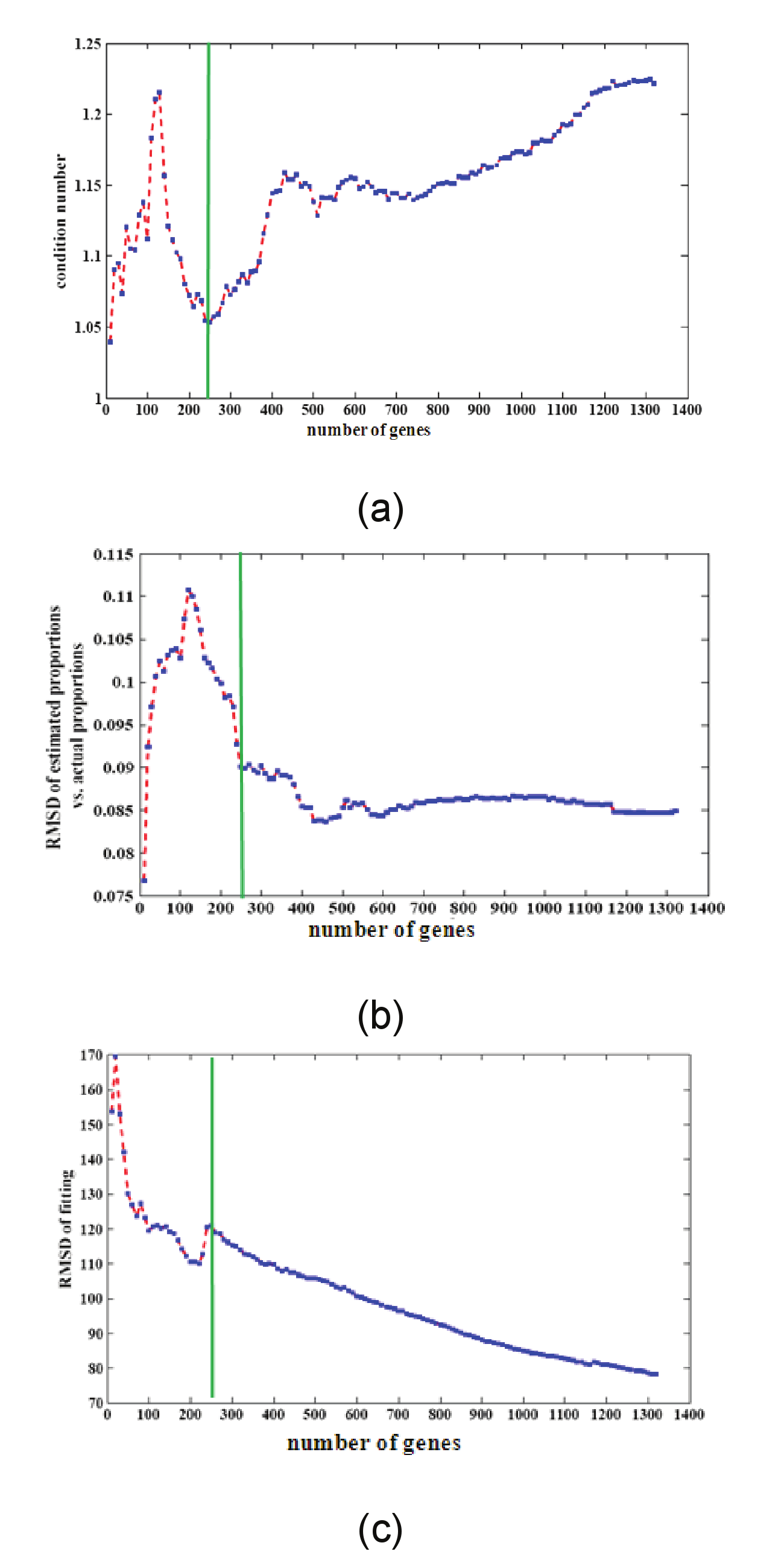

Supplement: Figure S1 — Condition number of signature basis matrix varies with number of probesets included. (a) Function of the condition number vs. the number of probesets from the gene signature was characterized in blood and breast mixture cell lines. The local minima of condition number is shown in green line and the corresponding number of genes was selected as the ‘optimal’ number of expression signature; (b) Root mean square deviation (RMSD) between the estimated fractions and the actual fractions showed clear patterns to support the ‘optimal’ number of expression signature selected in (a). To the right of the green line, the RMSD almost formed a horizontal line with minor oscillations, suggesting that increasing the number of genes would not increase the accuracy of the deconvolution estimates. (c) The RMSD of the fitting residual also had high correlation with the condition number. This correlation is weaker when selecting more than the ‘optimal’ number of genes (shown in green line here). (TIF) [file pone.0027156.s001.tif]

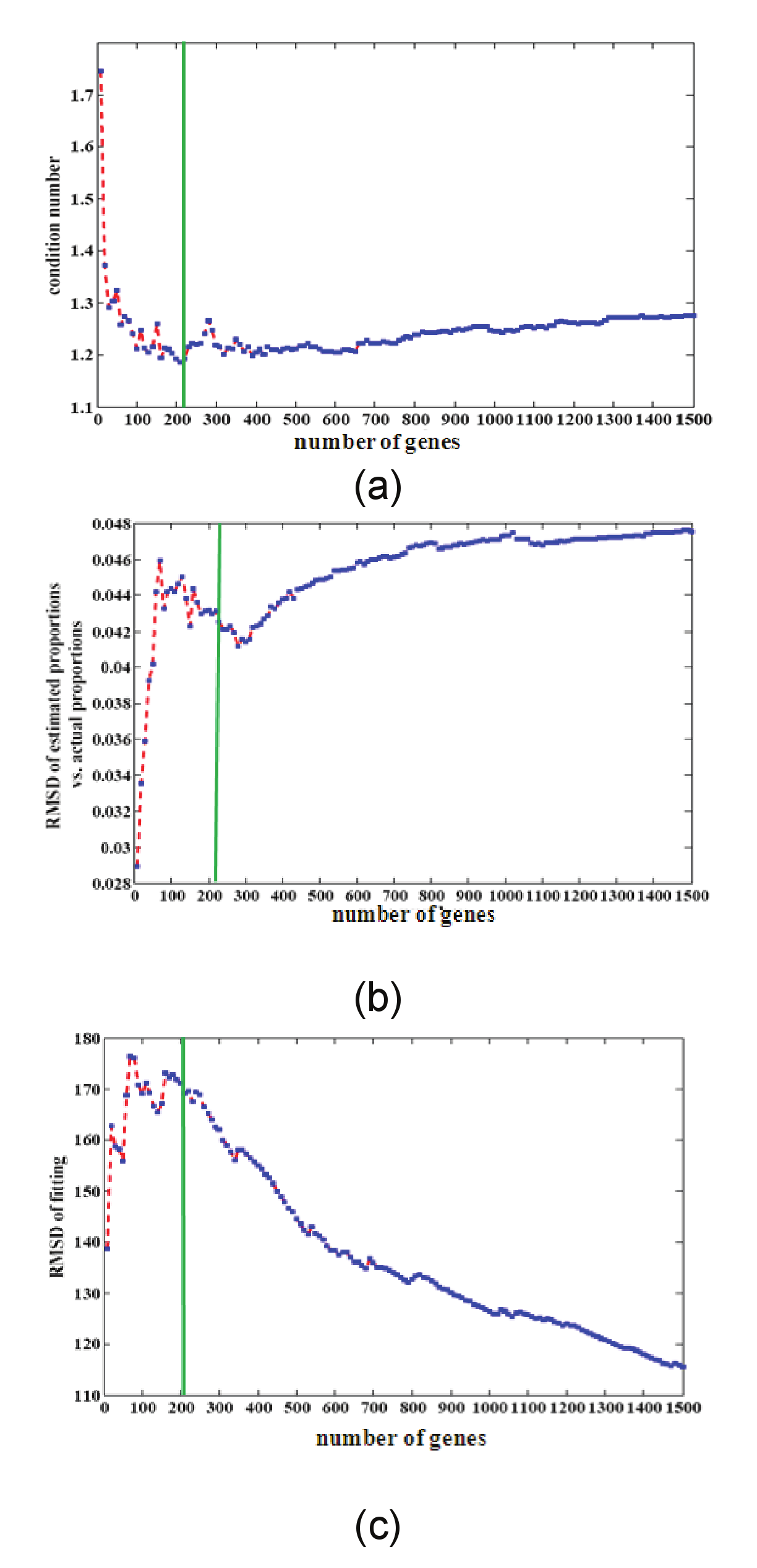

Supplement: Figure S2 — Condition number varies with the number of probesets included in liver/kidney signatures. (a) Function of the condition number vs. the number of probesets from the gene signature was characterized in rat liver and kidney mixture cell lines. The local minima of condition number is shown in green line and the corresponding number of genes was selected as the ‘optimal’ number of expression signature; (b) shows the relationship between the RMSD of the estimated fractions and the number of genes in basis matrix; (c) is the plot of the RMSD of the fitting residual vs. the number of genes in basis matrix. (TIF) [file pone.0027156.s002.tif]

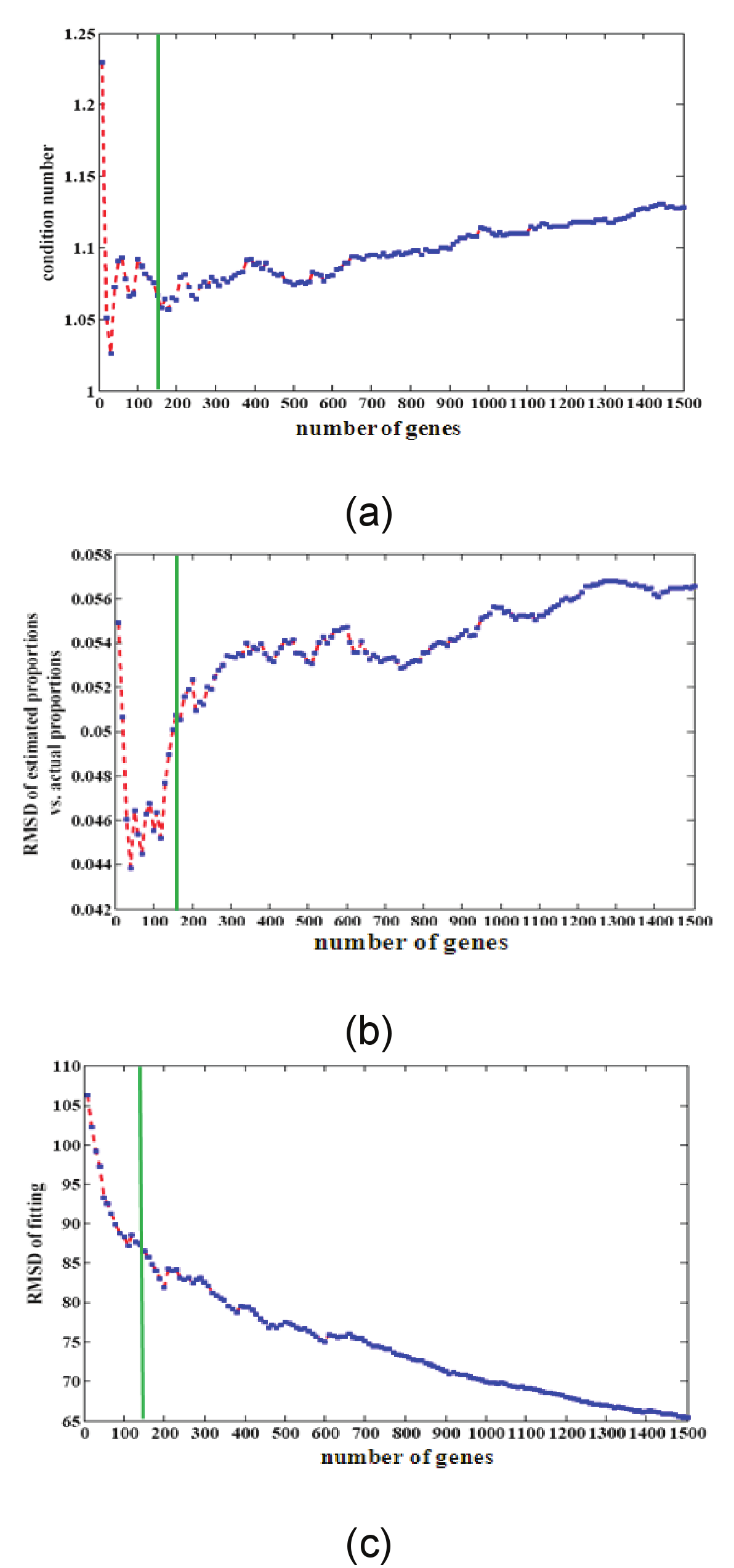

Supplement: Figure S3 — Condition number varies with the number of probesets included in liver/brain signatures. (a) Function of the condition number vs. the number of probesets from the gene signature was characterized in rat liver and brain mixture cell lines. The local minima of condition number is shown in green line and the corresponding number of genes was selected as the ‘optimal’ number of expression signature; (b) shows the relationship between the RMSD of the estimated fractions and the number of genes in basis matrix; (c) is the plot of the RMSD of the fitting residual vs. the number of genes in basis matrix. (TIF) [file pone.0027156.s003.tif]

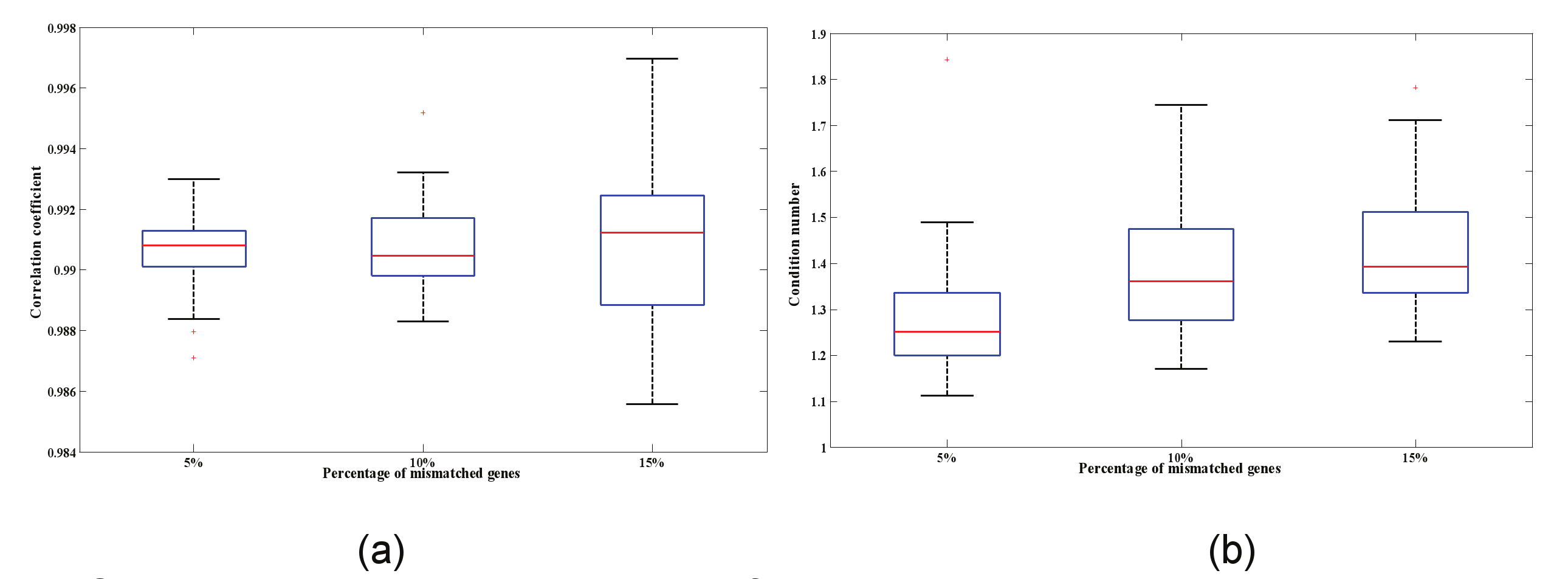

Supplement: Figure S4 — Stability of chosen signature matrix. (a) Boxplot displaying the stability of chosen signature matrix. The chosen signatures are distorted by randomly selecting 5, 10, or 15 percent of its genes and randomly modulating their values with 5 fold changes. The distribution of correlations between actual mixing fractions and fractions estimated using these signatures is depicted. (b) Condition number of the basis matrix with respect to the percentage of simulated differentially expressed genes in the basis matrix. (TIF) [file pone.0027156.s004.tif]
