# Supplementary material for: Optimal Deconvolution of Transcriptional Profiling Data Using Quadratic Programming with Application to Complex Clinical Blood Samples
Source: PLoS One. 2011 Nov 16;6(11):e27156. doi: 10.1371/journal.pone.0027156 (PMC3217948; doi:10.1371/journal.pone.0027156)
Supplement: Table S1 — Experimental design for rat liver vs . kidney microarray experiment. (DOC) [file pone.0027156.s005.doc]

**Table S1. Experimental design for rat liver *vs*. kidney microarray experiment.**

| Tissue Type | % Liver mRNA | % Kidney mRNA | # Replicates |
| --- | --- | --- | --- |
| pure | 0% | 100% | 6 |
| mixed | 25% | 75% | 6 |
| mixed | 75% | 25% | 6 |
| pure | 100% | 0% | 6 |
