# Supplementary material for: Optimal Deconvolution of Transcriptional Profiling Data Using Quadratic Programming with Application to Complex Clinical Blood Samples
Source: PLoS One. 2011 Nov 16;6(11):e27156. doi: 10.1371/journal.pone.0027156 (PMC3217948; doi:10.1371/journal.pone.0027156)
Supplement: Table S2 — Experimental design for rat brain vs. liver microarray experiment. (DOC) [file pone.0027156.s006.doc]

**Table S2. Experimental design for rat brain *vs*. liver microarray experiment**.

| Tissue Type | % Brain mRNA | % Liver mRNA | # Replicates |
| --- | --- | --- | --- |
| pure | 0% | 100% | 3 |
| mixed | 25% | 75% | 3 |
| mixed | 50% | 50% | 3 |
| mixed | 75% | 25% | 3 |
| pure | 100% | 0% | 3 |
