# Supplementary material for: Optimal Deconvolution of Transcriptional Profiling Data Using Quadratic Programming with Application to Complex Clinical Blood Samples
Source: PLoS One. 2011 Nov 16;6(11):e27156. doi: 10.1371/journal.pone.0027156 (PMC3217948; doi:10.1371/journal.pone.0027156)
Supplement: Table S4 — Leukocyte types used as the basis for whole blood deconvolution. (DOC) [file pone.0027156.s008.doc]

**Table S4. Leukocyte types used as the basis for whole blood deconvolution**.

| Resting helper T cells | Resting B cells | Plasma cells |
| --- | --- | --- |
| Activated helper T cells | Activated B cells | Resting NK cells |
| Resting cytotoxic T cells | BCR-ligated B cells | Activated NK cells |
| Activated cytotoxic T cells | IgM memory B cells | Monocytes |
|  | IgA/IgG memory B cells |  |
